# FXYD5 Is an Essential Mediator of the Inflammatory Response during Lung Injury

**DOI:** 10.3389/fimmu.2017.00623

**Published:** 2017-06-01

**Authors:** Patricia L. Brazee, Pritin N. Soni, Elmira Tokhtaeva, Natalia Magnani, Alex Yemelyanov, Harris R. Perlman, Karen M. Ridge, Jacob I. Sznajder, Olga Vagin, Laura A. Dada

**Affiliations:** ^1^Pulmonary and Critical Care Division, Feinberg School of Medicine, Northwestern University, Chicago, IL, United States; ^2^Department of Physiology, David Geffen School of Medicine, UCLA, Los Angeles, CA, United States; ^3^Veterans Administration Greater Los Angeles Healthcare System, Los Angeles, CA, United States; ^4^Division of Rheumatology, Feinberg School of Medicine, Northwestern University, Chicago, IL, United States

**Keywords:** alveolar epithelium, inflammation, FXYD5, acute lung injury, C-C chemokine ligand-2

## Abstract

The alveolar epithelium secretes cytokines and chemokines that recruit immune cells to the lungs, which is essential for fighting infections but in excess can promote lung injury. Overexpression of FXYD5, a tissue-specific regulator of the Na,K-ATPase, in mice, impairs the alveolo-epithelial barrier, and FXYD5 overexpression in renal cells increases C-C chemokine ligand-2 (CCL2) secretion in response to lipopolysaccharide (LPS). The aim of this study was to determine whether FXYD5 contributes to the lung inflammation and injury. Exposure of alveolar epithelial cells (AEC) to LPS increased FXYD5 levels at the plasma membrane, and FXYD5 silencing prevented both the activation of NF-κB and the secretion of cytokines in response to LPS. Intratracheal instillation of LPS into mice increased FXYD5 levels in the lung. FXYD5 overexpression increased the recruitment of interstitial macrophages and classical monocytes to the lung in response to LPS. FXYD5 silencing decreased CCL2 levels, number of cells, and protein concentration in bronchoalveolar lavage fluid (BALF) after LPS treatment, indicating that FXYD5 is required for the NF-κB-stimulated epithelial production of CCL2, the influx of immune cells, and the increase in alveolo-epithelial permeability in response to LPS. Silencing of FXYD5 also prevented the activation of NF-κB and cytokine secretion in response to interferon α and TNF-α, suggesting that pro-inflammatory effects of FXYD5 are not limited to the LPS-induced pathway. Furthermore, in the absence of other stimuli, FXYD5 overexpression in AEC activated NF-κB and increased cytokine production, while FXYD5 overexpression in mice increased cytokine levels in BALF, indicating that FXYD5 is sufficient to induce the NF-κB-stimulated cytokine secretion by the alveolar epithelium. The FXYD5 overexpression also increased cell counts in BALF, which was prevented by silencing the CCL2 receptor (CCR2), or by treating mice with a CCR2-blocking antibody, confirming that FXYD5-induced CCL2 production leads to the recruitment of monocytes to the lung. Taken together, the data demonstrate that FXYD5 is a key contributor to inflammatory lung injury.

## Introduction

The alveolar epithelium not only is responsible for gas exchange but also acts as a physical and immunological barrier for all inhaled substances and microbial products. Alveolar epithelial cells (AEC) also contribute to innate immunity by secreting cytokines and chemokines, which recruit phagocytic myeloid cells and other inflammatory cells to the site of infection (1–3). During lung inflammation, the interaction of monocyte chemoattractant protein C-C chemokine ligand-2 (CCL2) secreted by AEC with its receptor, CC chemokine receptor 2 (CCR2), results in cellular recruitment to the lung. Present in a subset of peripheral monocytes, CCR2 serves as marker for classical monocyte inflammation (4–7). Recruitment of circulating monocytes to tissues is essential for effective control and clearance of infections, but if not controlled, it can become harmful, contributing to disease progression.

The alveolar epithelium is comprised of large flat type I alveolar (ATI) epithelial cells and cuboidal type II alveolar (ATII) epithelial cells. Both ATI and ATII cell types have important roles in airway surveillance through the initial recognition of microbial pathogens and bacterial toxins by various pattern recognition receptors (PRR) such as toll-like receptors (TLR) and nod-like receptors to activate the host defense (8). Lipopolysaccharide (LPS), a glycolipid of the outer membrane of Gram-negative bacteria is a major cause of morbidity and mortality in humans (9–11). Acute exposure to LPS increases cytokine release and disrupts the alveolo-capillary barrier, resulting in pulmonary edema and the recruitment of inflammatory cells into the lung (12–15). The response to LPS is initiated by interaction with TLR4 in association with the accessory proteins MD-2 and CD-14 (10, 16, 17). TLR4 is constitutively expressed in primary alveolar type II cells as well as in the adenocarcinoma cell line A549 (18). Exposure of lung epithelial cells to LPS leads to the activation of the NF-κB family of transcription factors, which in turn directs the expression of pro-inflammatory mediators (16, 19).

FXYD proteins, named after an invariant FXYD sequence, were first described as tissue-specific modulators of Na,K-ATPase activity (20–26). This family contains seven integral membrane proteins that interact with the Na,K-ATPase and regulate its function in a tissue-specific manner (27, 28). FXYD5, also known as dysadherin, is not only involved in the regulation of Na,K-ATPase activity (29, 30) but also acts as a tumorigenic protein when overexpressed (31, 32). Its expression is elevated in metastatic tumors, suggesting FXYD5 as an oncogenic marker (32–38). FXYD5 is also expressed in normal tissues, including the alveolar epithelium (24, 26, 29, 30, 39, 40). In cancer cells, CCL2 has been identified as a mediator of FXYD5 effects on cell migration (41). In these cells, FXYD5 has been shown to regulate CCL2 expression through the activation of the NF-κB signaling pathway.

Several publications have suggested a role of FXYD5 in the regulation of inflammation. In AEC, we have described that *in vivo* overexpression of FXYD5 impairs the interaction between Na,K-ATPase subunits in neighboring cells, disrupting the alveolar barrier (26), which might contribute to the recruitment of inflammatory cells into the alveolar compartment. Also, overexpression of FXYD5 in normal kidney epithelial cells increases the inflammatory response to LPS in a tumor necrosis factor α (TNF-α) receptor-dependent manner and the levels of FXYD5 are increased in lungs after treatment of mice with LPS (30). Supporting a role for FXYD5 in inflammatory diseases, the expression levels of FXYD5 are elevated in the lungs of patients with acute lung injury (42). However, whether endogenous FXYD5 plays a role in the epithelial inflammatory response remains mostly unknown. Here, using *in vivo* and *in vitro* models, we investigated the mechanism by which the increase of FXYD5 in AEC contributes to lung inflammation and injury.

## Materials and Methods

### Reagents

Chemical and cell culture reagents were purchased from Sigma-Aldrich or Corning Life Sciences unless stated otherwise. LPS from Escherichia coli 0111:B4 was from Sigma-Aldrich.

### Cell Culture

Mouse lung epithelial MLE-12 and human epithelial A549 cells (ATCC) were grown and maintained as previously described (43, 44).

### LPS-Induced Lung Inflammation and Injury Model

Mice were provided with food and water *ad libitum*, maintained on a 14-h-light–10-h-dark cycle, and handled according to National Institutes of Health guidelines and an experimental protocol approved by the Northwestern University Institutional Animal Care and Use Committee. C57BL/6 mice (10–12 weeks of age) were given intratracheal instillation of LPS (3 mg/kg body weight) for up to 24 h as we previously described (45). Bronchoalveolar lavage fluid (BALF) was obtained through a 20-gage angiocath ligated into the trachea through a tracheostomy (26). A total of 1-ml of PBS was instilled into the lungs and then aspirated three times. BALF was collected for cell counts, protein quantification, and cytokine determination as we previously described (30, 46). RNA was isolated from lung peripheral tissue using an RNeasy kit (QIAGEN) and reverse transcribed using qScript cDNA synthesis (Quanta Biosciences). Quantitative PCRs were set up using iQ SYBR Green Super mix (Bio Rad). Data were normalized to the abundance of L19 mRNA. The primers for FXYD5, CCL2, GAPDH, and L19 were: FXYD5 5′ CAT CCT ACA TTG AAC ATC CA 3′ and 5′ TGA GAC AAC TGC CTA CAC 3′; L19 5′ AGC CTG TGA CTG TCC ATT C 3′ and 5′ ATC CTC ATC CTT CTC ATC CAG 3′; CCL2 5′ CCT GTC ATG CTT CTG GGC CTG C 3′ and 5′ GGG GCG TTA ACT GCA TCT GGC TG 3′; and GAPDH 5′ AAC TTT GGC ATT GTG GAA GGG CTC 3′ and 5′ TGG AAG AGT GGG AGT TGC TGT TGA 3′. Proteins were determined in cell lysates or total membranes as we previously described (26, 43).

### Lentivirus Instillation

To knock down mouse FXYD5 protein *in vivo* in lung, we generated the VSVG pseudotyped lentiviruses (10^9^–10^10^ TU/ml) expressing mouse FXYD5 shRNA and non-silencing shRNA as control (47, 48) (provided by DNA/RNA Delivery Core, SDRC, Northwestern University, Chicago, IL, USA). For lentivirus packaging, 293T packaging cells (Gene Hunter Corporation) were transiently transfected using Transit-2020 reagent (Mirus) with the following vectors: second generation packaging vectors psPAX2 and pMD2.G (Addgene) and third generation lentiviral expression vector pLKO (Sigma). The pLKO vectors used encoded two specific shRNAs against mouse FXYD5 (Cat# TRCN0000079348, sense: CCTCCAAACTACACCAACTCA; and Cat# TRCN0000079352, sense: GTGCTGTTCATCACGGGAATT), and a non-silencing control shRNA (Cat# SHC002) (all from Sigma). FXYD5 shRNA and control non-silencing shRNA viruses were intratracheally instilled in mice in a volume of 50 µl. FXYD5 silencing was confirmed by RT-qPCR and Western blot analysis as described above.

### Adenoviral Infection

*CCR2^−/−^* mice were purchased from Jackson Laboratories (49). WT C57BL/6 or *CCR2^−/−^* mice at 8–12 weeks of age were infected with Ad-mCherry-HA-FXYD5 (Ad-FXYD5; 1 × 10^9^ plaque-forming units (pfu)/animal) in 50% surfactant vehicle as previously described (30, 50) and housed in a containment facility. After 72 h, BALF was collected and used as described above. Control adenovirus (Ad-Null) was purchased from Viraquest, Inc. Cells were infected with Ad-Null or Ad-FXYD5 20 pfu/cell as previously described (26).

### Analysis of Cytokines and Chemokines

The concentration of CCL2/MCP-1 (Affymetrix), TNF-α (Affymetrix), and IL-6 (Life Technologies) in the BALF or cell culture supernatants were quantified by ELISA following the manufacturer’s instructions.

### *In Vitro* Treatment of AEC and siRNA Transfection

MLE-12 or A549 cells were transfected with 120 pmol of mouse or human FXYD5 siRNA duplex (final concentration 100 µM) (Santa Cruz Biotechnology), respectively, using Lipofectamine RNAiMAX (Invitrogen). A non-silencing negative control siRNA was purchased from Santa Cruz Biotechnology. Experiments were performed 24 h after transfection. Cells were starved for 2 h by incubation in culture media containing 2.5% fetal bovine serum and treated with LPS (100 ng/ml) for the indicated times. Supernatants were collected for cytokine analysis and cells were biotinylated by the membrane-impermeable biotinylation reagent where indicated; cells lysates, total membranes, or surface biotinylated proteins were isolated for SDS-PAGE and immunoblot analysis as previously described (26, 43). The following mouse monoclonal antibodies were used: HA (Biolegend clone 16B12 #901502; 1:1,000), pIKBα (Cell Signaling Technology #9246; 1:500), IKBα (Cell Signaling Technology #4814; 1:500), E-cadherin (E-cad) (BD Biosciences #610182, 1:2,500 dilution). The following polyclonal antibodies were used: FXYD5 (Sigma-Aldrich #HPA010817, 1:1,000 and M178 from Santa Cruz Biotechnology #98247, 1:200), and β-actin (Cell Signaling Technology #4967, 1:1,000). Immunoblots were quantified by densitometry using Image J 1.46r (National Institutes of Health, Bethesda, MD). Where indicated, surface biotinylated proteins were treated with *O*-glycosidase and Neuraminidase Bundle according to the manufacturer’s instructions (New England Biolabs, Inc.) prior to loading on SDS-PAGE as we previously described (26).

Interferon α (IFN-α, Biolegend) and TNF-α (Biolegend) were added to A549 cells for up to 24 and 2 h, respectively, as described for LPS treatment.

### Flow Cytometry and Cell Sorting

Myeloid populations from whole lung were isolated and defined as previously described (51). Briefly, perfused lungs were inflated with digestion buffer (1 mg/ml of Collagenase D and 0.1 mg/ml DNase I, both from Roche) and coarsely minced with scissors before processing in C-tubes (Miltenyi) with a GentleMACS dissociator (Miltenyi), according to the manufacturer’s instructions. Homogenate was passed through 40-µm nylon mesh to obtain a single-cell suspension and subjected to red blood cell lysis (BD Pharm Lyse, BD Biosciences). Live cells were counted using a Countess cell counter (Invitrogen) by trypan blue exclusion.

Cells were then stained with the following cocktail: CD45-FITC (eBioscience #11-0451-81, 0.1 μg/μl), MHCII-PerCPCy5.5 (Biolegend #107626, 0.01 μg/μl), Ly6C-eFluor450 (eBioscience #348-5932-80, 0.02 μg/μl), CD24-APC (eBioscience #317-0242-80, 0.01 μg/μl), Ly6G-Alexa700 (BD Bioscience #561236, 0.04 μg/μl), NK1.1-Alexa700 (BD Bioscience #560515, 0.06 μg/μl), CD11b-APCcy7 (Biolegend #101225, 0.02 μg/μl), CD64-PE (Biolegend #139303, 0.02 μg/μl), SiglecF-PECF594 (BD Bioscience #562757, 0.02 μg/μl), CD11c-PEcy7 (BD Bioscience #561022, 0.02 μg/μl). Multicolor flow cytometry was performed with an LSR Fortessa using DIVA software (BD Biosciences) and the following gating outlined below and in Figure 5. FlowJo software version 10.0.8 (FlowJo, LLC) was used for all compensation and data analysis.

After excluding doublets and dead cells, myeloid cells were identified using the pan-hematopoietic marker CD45 (51). As shown in Figure 5, the CD45^+^ population was then separated into Ly6G/NK1.1^−^ and Ly6G/NK1.1^+^ populations using a shared channel to pull out NK cells (NK1.1^+^ CD11b^hi^ CD24^hi^) and neutrophils (Ly6G CD11b^int^ CD24^int^). The Ly6G/NK1.1^−^ population was further divided based on SiglecF and CD11c expression to identify alveolar macrophages (SiglecF^hi^ CD11c^hi^) and eosinophils (SiglecF^hi^ CD11c^low^). From the remaining SiglecF^low^ CD11c^low^ group, a CD11b^hi^ population was then selected and segregated based on MHCII expression. Within the MHCII^low^ cluster, cells could be defined as classical monocytes (Ly6C^hi^) or non-classical monocytes (Ly6C^low^). Alternatively, interstitial macrophages (IMs) were identified as MHCII^hi^ CD64^hi^ CD24^low^.

Mouse ATII cells (mATII) were isolated and defined as previously described (52). Briefly, whole lung was subjected to enzymatic and manual digestion to obtain a single cell suspension. Cells were then stained with Epcam (eBioscience #17-5791-80, 0.1 μg/ml), CD45, CD31-PE (eBioscience #12-0311-81, 0.1 μg/ml), and MHCII-eFlour450 (eBioscience #48-5321-82, 0.1 μg/ml). mATII cells were identified as CD45^−^ EpCAM^+^, CD31^−^ and sorted on a BD FACSAria 5-laser.

### Fluorescent Staining and Confocal Microscopy

Isolated mATII cells were plated on glass-bottom dishes (MatTek corporation), fixed by incubation with 3.75% formaldehyde in PBS for 15 min at 37°C, and actin filaments were visualized using fluorescein phalloidin (Thermo Fisher Scientific) as described previously (53). Confocal microscopy images of mCherry-tagged FXYD5 and stained actin filaments were acquired using a Zeiss LSM 510 laser scanning confocal microscope and ZEN 2009 software (Carl Zeiss MicroImaging GmbH).

### Anti-CCR2 Antibody Treatment

Mice were injected retro-orbitally with 6 μg/100 μl of anti-CCR2 monoclonal antibody (clone MC-21) in PBS (49) 48 h after adenoviral instillation. Mice were sacrificed after 24 h and BALF was obtained as described above.

### Statistical Analysis

Data are expressed as mean ± SD. For comparisons between two groups, significance was evaluated by Student’s *t*-test, and when more than two groups were compared, one-way ANOVA was used followed by the Dunnett’s or Sidak test using GraphPad Prism 7.02 software.

## Results

### The Increase in FXYD5 Is Required for the Secretion of Inflammatory Mediators by the AEC in Response to LPS

**Figure 1:**
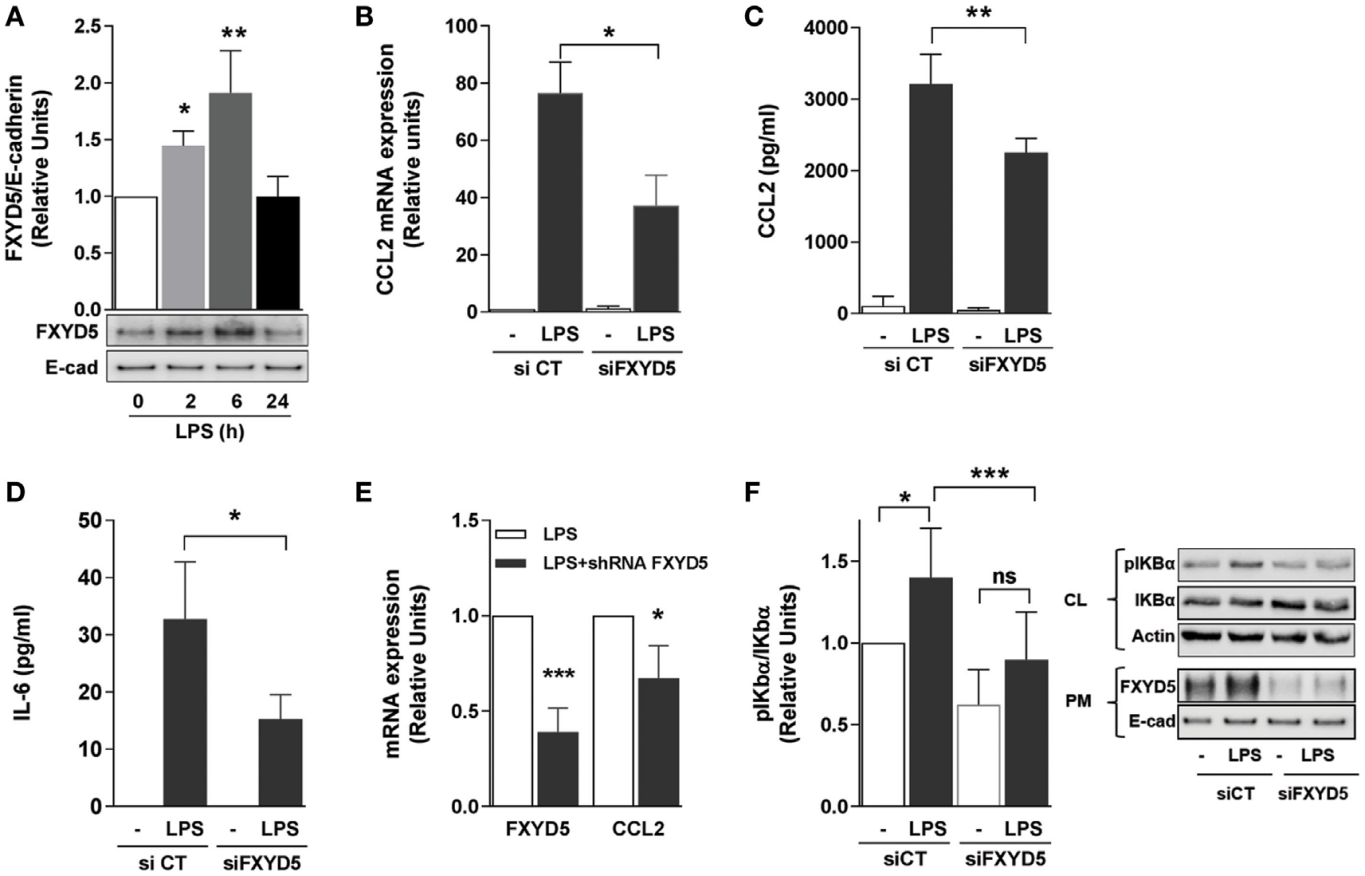
**FXYD5 plays a role in LPS-induced inflammatory response by activating the NF-κB signaling pathway in alveolar epithelial cells**. **(A)** MLE-12 cells were treated with 100 ng/ml LPS for the indicated period of time, PM proteins were isolated after cell surface labeling with biotin and characterized by immunoblot with an anti-FXYD5 specific antibody. Densitometric quantification of immunoblots of FXYD5 in relation to E-cad is shown (*n* = 3). **(B)** MLE-12 were cells transfected with a FXYD5-specific siRNA and 24 h later treated with LPS for 6 h. CCL2 mRNA was measured by RT-qPCR *n* = 4. **(C,D)** MLE-12 cells were treated like in B, culture media was collected, and CCL2 **(C)** and IL-6 **(D)** were determined by ELISA (*n* = 5). **(E)** FXYD5 was silenced in isolated mATII cells with shFXYD5 for 72 h and then treated with LPS for 6 h. FXYD5 and CCL2 mRNA were measured by RT-qPCR (*n* = 3). **(F)** A549 cells were treated as in B and cell lysate (CL) and PM proteins were isolated after cell surface labeling with biotin. Densitometric quantification (left panel) of immunoblots (right panel) of pIκBα in relation to total IκBα is shown (*n* = 6). Values of PBS-treated controls were normalized to 1. Bars represent means ± SD. Statistical significance was analyzed by one way ANOVA and Dunnetts’s **(A)** or Sidak’s multiple comparison test **(B–D,F)** or Student’s *t*-test **(E)**. *p ≤ 0.05; **p ≤ 0.01; ***p ≤ 0.001; ns, non-significant.

Alveolar epithelial cells produce the first wave of cytokines, which trigger local and systemic inflammatory responses (54). We have reported that overexpression of FXYD5 in normal kidney epithelial cells increases the inflammatory response to LPS (40) and that overexpression of FXYD5 in the mouse alveolar epithelium increases alveolar epithelial permeability (26). Moreover, treatment of mice with LPS increased the level of FXYD5 in lungs (30). However, whether endogenous FXYD5 plays a role in the generation of an alveolar epithelial inflammatory response to LPS has not been studied. To determine whether LPS modulates FXYD5 levels in AEC, MLE-12 cells were treated with LPS for up to 24 h and cell culture media, cell lysates, and surface biotinylated plasma membrane (PM) proteins were collected. In the PM fraction of MLE-12 cells, FXYD5 was detected as a 60–70 kDa band (Figure 1A), suggesting that the plasmalemma-located FXYD5 is heavily *O*-glycosylated in these cells similar to that found in A549 cells (26). An additional 25 kDa band was seen in MLE-12 cell lysates (not shown) that represents the intracellular immature unglycosylated or less glycosylated fraction of FXYD5. LPS time dependently increased PM level of FXYD5 in MLE-12 cells with a peak at 6 h (Figure 1A). CCL2, which is abundantly produced by ATII cells, plays an important role in the local regulation of inflammatory processes (55). As expected, treatment of MLE-12 cells for 6 h with LPS strongly stimulated CCL2 mRNA synthesis as well as the secretion of CCL2 and IL-6 into the culture media compared with untreated controls (Figures 1B–D). Silencing of FXYD5 in those cells with a specific siRNA prevented the LPS-stimulated increase in the transcription of CCL2 and secretion of both cytokines as compared with a control siRNA (Figures 1B–D). In isolated primary mouse ATII, infection with lentivirus coding for specific shRNA FXYD5 prevented the LPS-stimulated increase in FXYD5 and CCL2 mRNA by 62 and 30%, respectively (Figure 1E).

Next, we investigated the signaling pathway by which the increase in FXYD5 regulates cytokine production. The dominant pathway triggered by PRR activation is the canonical NF-κB pathway. The NF-κB complex comprises IκB (inhibitor of NF-κB) bound to two proteins, p50 and p65; when not stimulated, the complex resides in the cytoplasm. Different stimuli lead to phosphorylation and degradation of IκB removing inhibitory effects and allowing the translocation of active NF-κB, the p50-p65 heterodimer, to the nuclei (56). Nuclear translocation triggers the expression of over 150 genes, including those encoding cytokines (57). We analyzed whether FXYD5 promotes the secretion of cytokines *via* the NF-κB signaling pathway by assessing the phosphorylation of one of IκB proteins, IκBα. In A549 cells, 6 h of LPS treatment led to an increase in FXYD5 at the PM similar to the one observed in MLE-12 (Figure 1F). A549 cells were transfected with siRNA specific for FXYD5, 24 h later stimulated with LPS for 6 h, and phosphorylation of IκBα was assessed in cell lysates. LPS treatment increased the IκBα phosphorylation, which was prevented by FXYD5 silencing (Figure 1F). Taken together, the results indicate that FXYD5 is required for the NF-κB-dependent secretion of inflammatory cytokines and chemokines induced by LPS, suggesting that FXYD5 is an important mediator of the pro-inflammatory response of AEC to LPS.

### Increased FXYD5 Is Sufficient to Induce AEC Secretion of Inflammatory Mediators

**Figure 2:**
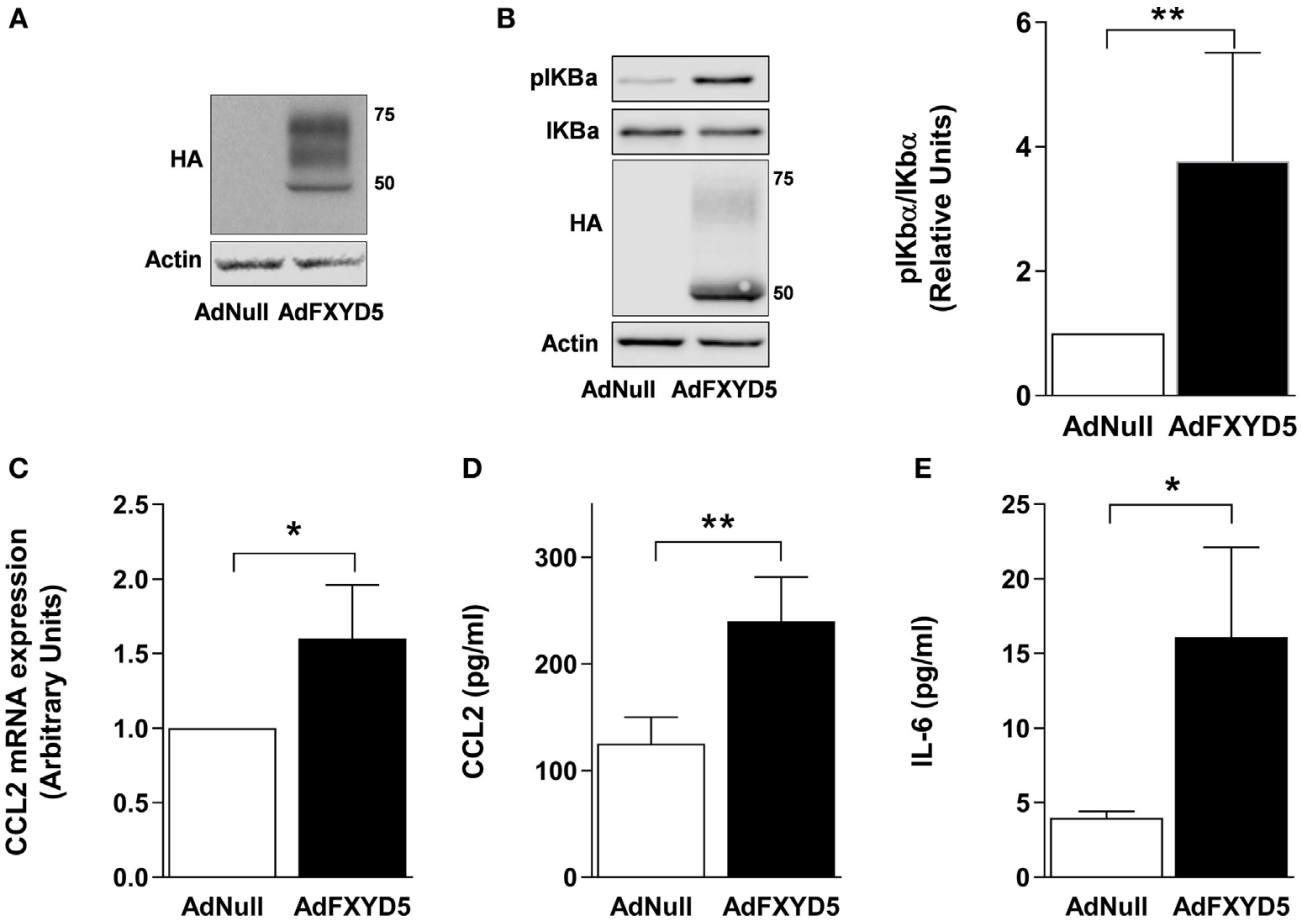
**Overexpression of FXYD5 promotes the secretion of inflammatory cytokines by activating the NF-κB signaling in alveolar epithelial cells**. **(A)** MLE-12 cells were incubated with Ad-Null or Ad-FXYD5 as described in Section “Materials and Methods.” The expression of FXYD5 was determined in the CL by Western blot with an HA antibody. **(B)** A549 cells were treated as in **(A)** and CL was isolated. Left panel: representative immunoblots. Right panel: densitometric quantification of pIκBα in relation to total IκBα. The expression of FXYD5 was determined with an HA antibody (*n* = 4). **(C)** MLE-12 cells were treated as in **(A)** and CCL2 mRNA was quantified by RT-qPCR (*n* = 3). **(D–E)** MLE-12 cells were treated as in **(A)**, culture media was collected, and CCL2, *n* = 5 **(D)** and IL-6, *n* = 5 **(E)** were determined by ELISA. Values of AdNull-treated controls were normalized to 1. Bars represent means ± SD. Statistical significance was analyzed by unpaired Student’s *t-*test. *p ≤ 0.05; **p ≤ 0.01.

Previous studies in breast cancer cells have demonstrated that FXYD5 knockdown decreases, while FXYD5 overexpression increases, both the NF-κB-responsive promoter activity and CCL2 production in cancer cells (41). To determine whether the increase in FXYD5 in AEC induces the NF-κB-dependent secretion of pro-inflammatory mediators, we infected cells with Ad-FXYD5, and 40 h after infection, determined the expression of FXYD5 (Figure 2A), phosphorylation of IκBα (Figure 2B), cellular levels of CCL2 mRNA (Figure 2C), and CCL2 and IL-6 levels in the culture media (Figures 2D,E). Expression of exogenous FXYD5 stimulated IκBα phosphorylation, the synthesis of CCL2 mRNA, and the release of CCL2 and IL-6 by AEC, suggesting that the increase in FXYD5 is sufficient to induce the inflammatory response in AEC.

### Increased FXYD5 in AEC Contributes to the Inflammatory Response to LPS *In Vivo*

**Figure 3:**
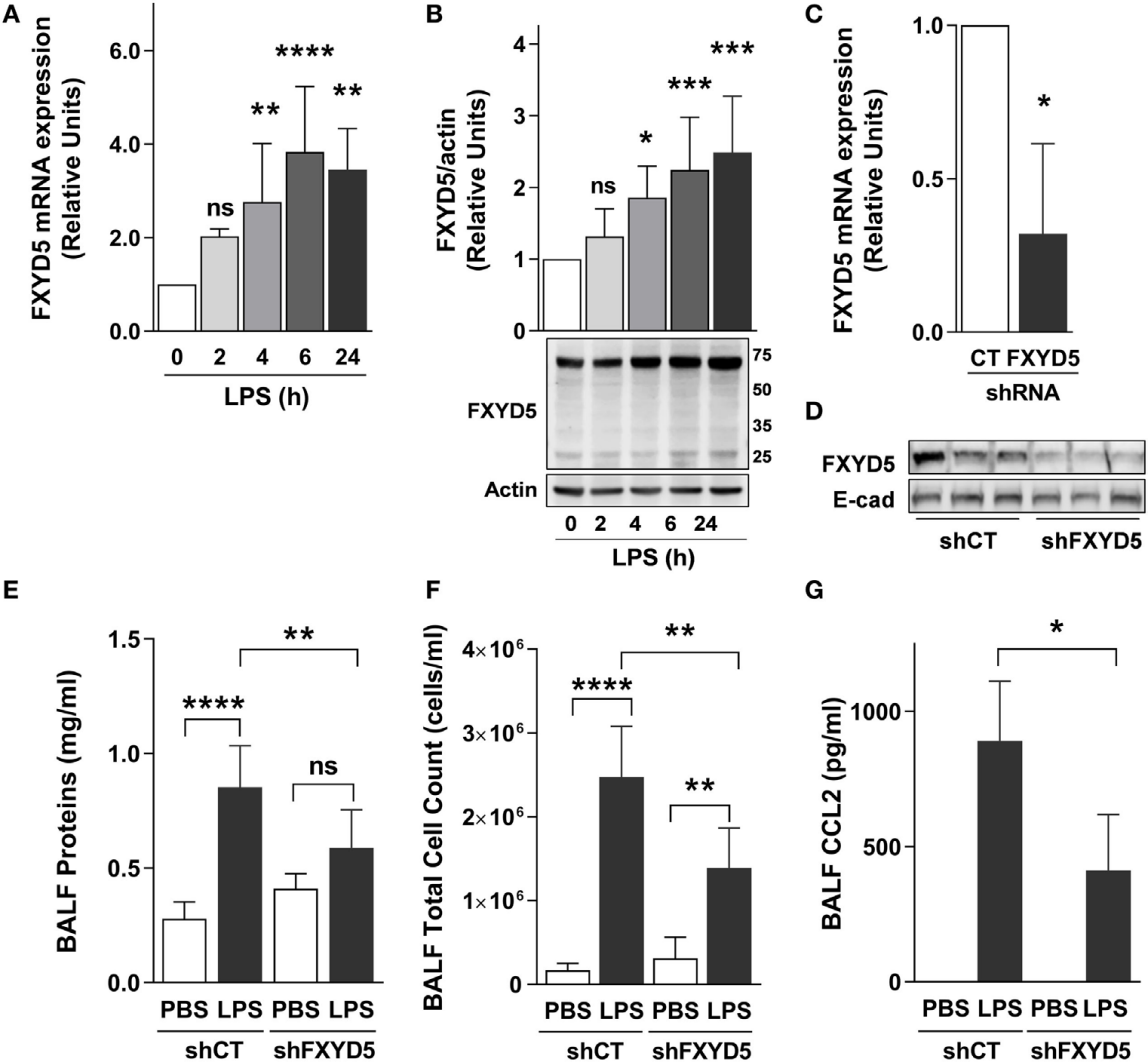
**Increased levels of FXYD5 are required for LPS-induced lung inflammation**. **(A)** LPS was instilled to mice for the indicated times and the levels of FXYD5 mRNA were determined by RT-qPCR in lung peripheral tissue (*n* = 8). **(B)** Mice were treated as in A and FXYD5 was determined in lung peripheral tissue cell lysates by Western blot. Bars indicate densitometric quantification of plasma membrane FXYD5 (top band) in relation to actin (*n* = 8). **(C,D)** Control (CT) or shFXYD5 lentiviral constructs were instilled into mice. Silencing was assessed by measuring FXYD5 mRNA by RT-qPCR **(C)**. *n* = 4 or protein abundance in lung peripheral tissue total membranes **(D)**. Representative immunoblot showing the abundance of FXYD5, E-cadherin was used as a loading control *n* = 6. **(E–G)** Mice treated as in C were given LPS for 6 h and BALF was obtained. Proteins **(E)**, total cells **(F)**, and CCL2 **(G)** were determined as described in Section “Materials and Methods.” Values of PBS-treated controls were normalized to 1. Bars represent means ± SD. Statistical significance was analyzed by one way ANOVA and Dunnetts’s **(A,B)** unpaired Student’s *t*-test **(D)** or Sidak’s multiple comparison test **(E,F)**. *p ≤ 0.05; **p ≤ 0.01; ***p ≤ 0.001.

To determine whether FXYD5, which is abundantly expressed in ATII cells (26), contributes to the inflammatory response in LPS-induced acute lung injury, we performed intratracheal instillation of LPS into mice and measured FXYD5 mRNA and protein levels in lung peripheral tissue after 2, 4, 6, and 24 h. Administration of LPS time dependently increased the level of FXYD5 mRNA with a peak after 6 h of instillation (Figure 3A). In mouse lung peripheral tissue lysates, FXYD5 was detected by Western blot in two bands, a major band at 60–70 kDa and a minor band at 25 kDa (Figure 3B). Only the 60–70 kDa fraction of FXYD5 is seen in surface biotinylated fraction in MLE-12 cells (Figure 1B), indicating that the 60–70 kDa in mouse lung lysates corresponds to the mature heavily glycosylated FXYD5 residing at the PM. LPS increased the abundance of both forms in a time-dependent manner. To assess whether the inflammatory response elicited by LPS in the lung is dependent on the presence of FXYD5, we silenced FXYD5 by instillation of lentiviral particles coding for shFXYD5, which decreased FXYD5 expression in the peripheral tissue by ~70% (Figures 3C,D). Treatment with LPS increased the concentration of proteins in the BALF (a measure of the permeability of the alveolo-capillary barrier) and total cell count in BALF (a measure of inflammatory cell recruitment to the lung) (Figures 3E,F). A decrease in FXYD5 in the lung peripheral tissue lowered the concentration of proteins in the BALF after LPS treatment as compared with control mice exposed to LPS (Figure 3E). Also, BALF from mice with silenced FXYD5 contained fewer inflammatory cells and reduced CCL2 after LPS treatment than that obtained from sh-control-treated mice (Figures 3F,G), suggesting that FXYD5 contributes to the LPS-induced production of CCL2 and the recruitment of inflammatory cells into the lung.

**Figure 4:**
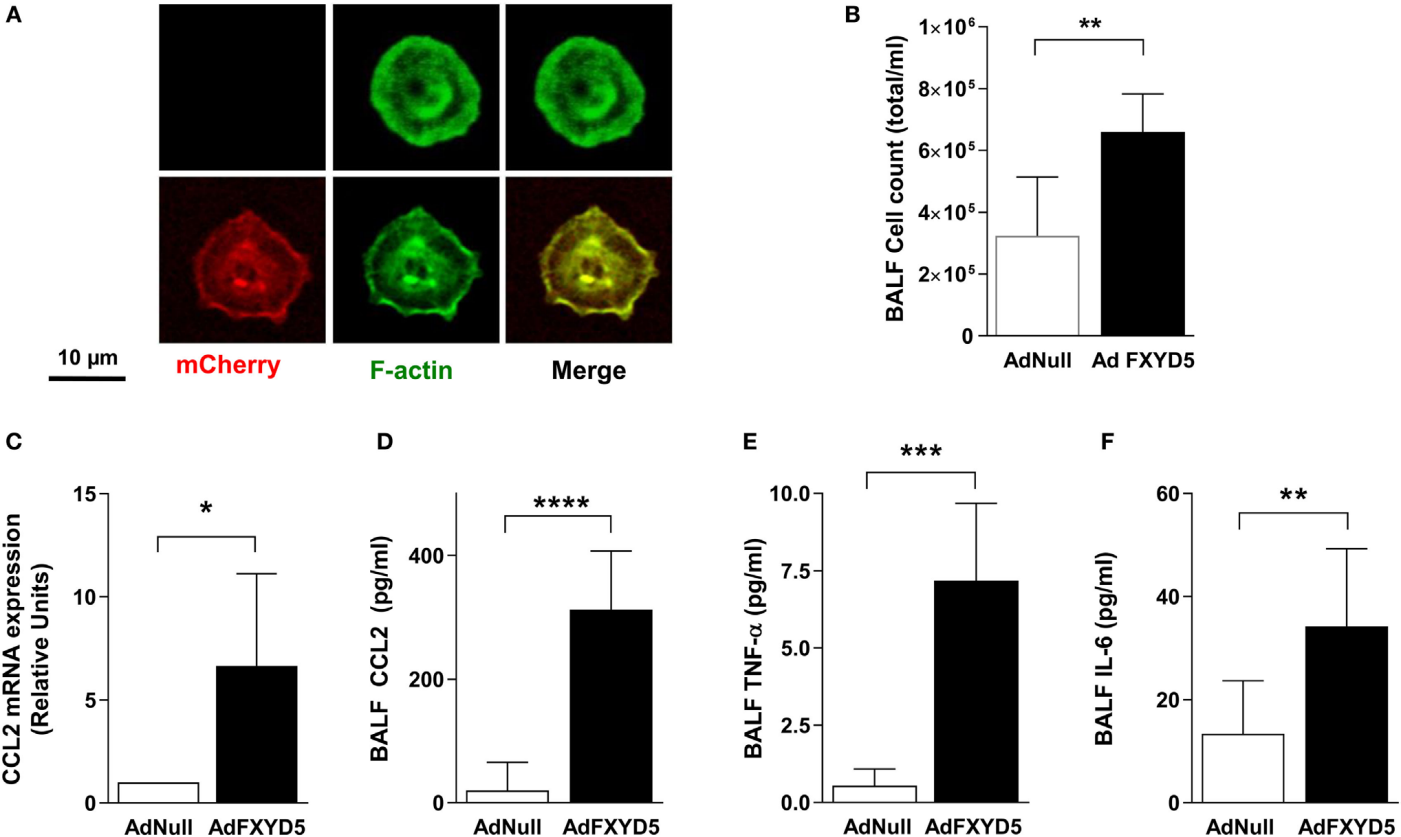
**Increased levels of FXYD5 are sufficient to induce lung inflammation**. Mice were instilled with adenoviruses encoding m-Cherry-HA-FXYD5 (AdFXYD5) or an empty control (AdNull) and 72 h later ATII cells, BALF or lung peripheral tissue were isolated. **(A)** Confocal microscopy analyses of FACS-sorted mice ATII cells. Red m-cherry fluorescence reflects the expression of FXYD5. Green fluorescence shows actin filaments used to visualize cells. **(B)** Total cell count in BALF. **(C)** CCL2 mRNA was determined by RT-qPCR in lung peripheral tissue. **(D–F)** CCL2, TNF-α, and IL-6 were determined by ELISA in BALF. Values of Ad-Null-treated controls were normalized to 1. Bars represent means ± SD *n* ≤ 5. Statistical significance was analyzed by unpaired Student’s *t*-test. *p ≤ 0.05; **p ≤ 0.01; ***p ≤ 0.001.

To determine whether the relationship between elevated FXYD5 and inflammation is causal, we studied the effects of intratracheal administration of an endotoxin-free adenoviral construct coding for mouse mCherry-HA-FXYD5 (Ad-FXYD5) or an empty adenovirus (Ad-Null) to mice (26). ATII cells from infected mice were isolated by flow-cytometry as CD45^−^ CD31^−^Ep-Cam^+^ cells. The expression of exogenous FXYD5 in ATII cells was evident from the red fluorescence of the mCherry tag present in this construct (Figure 4A). Instillation of Ad-FXYD5 increased total cell number in BALF (Figure 4B) as compared with mice infected with Ad-Null. Moreover, in agreement with our *in vitro* data, FXYD5 overexpression in mice increased the levels of CCL2 mRNA (Figure 4C) and the secretion of CCL2, TNF-α, and IL-6 into the alveolar space (Figures 4D–F).

### FXYD5 Induces the Recruitment of Different Subsets of Myeloid Cells to the Lung

**Figure 5:**
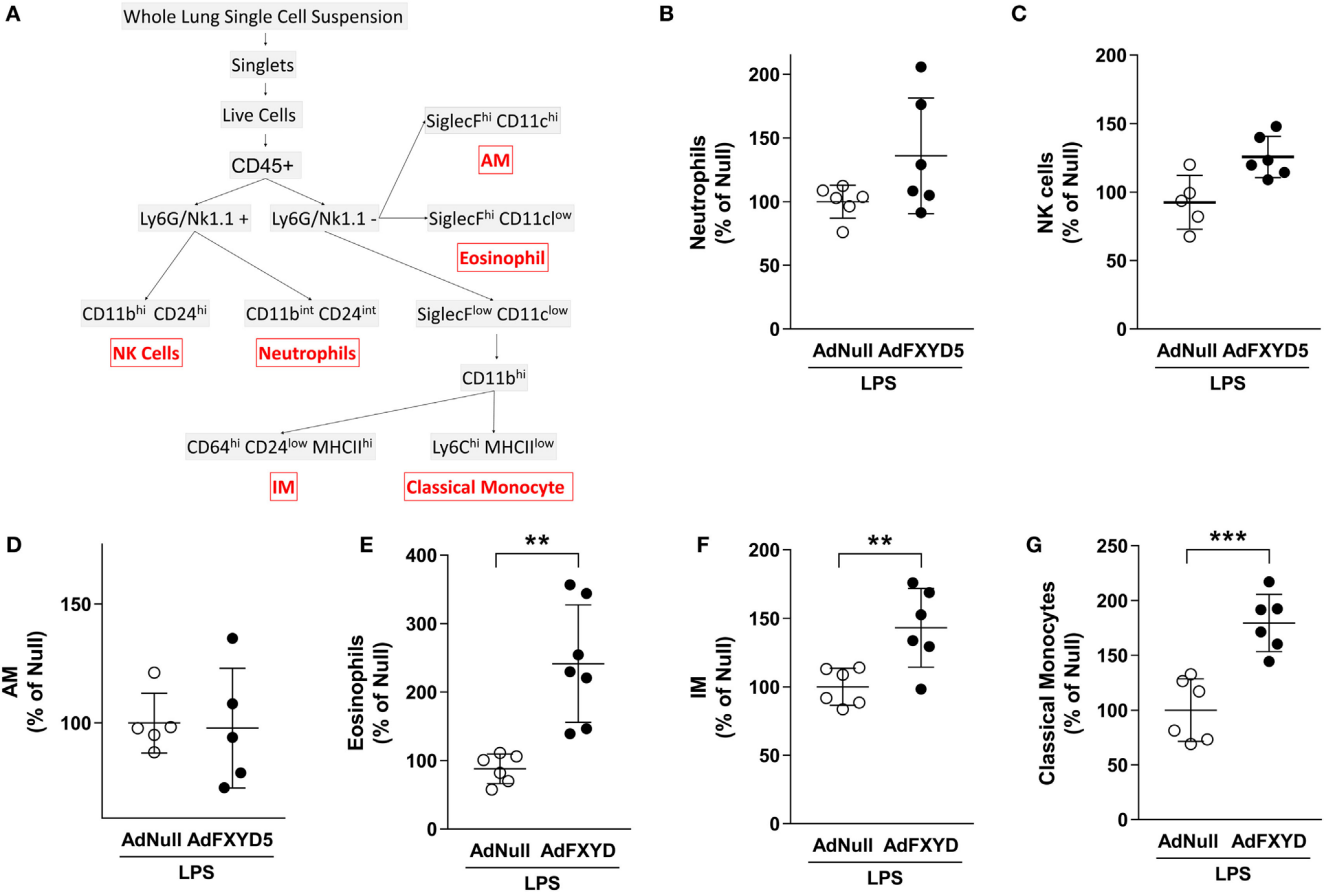
**FXYD5-induced changes in myeloid-cell subsets in mouse lungs during LPS-induced lung injury**. **(A)** Gating strategy used to identify myeloid-cell subsets in the normal mouse lung. **(B–G)** Mice were instilled with Ad-FXYD5 or Ad-Null and treated with LPS for 12 h. Cells were isolated from enzymatically digested mouse lungs. Changes of myeloid-cell subsets in Ad-FXYD5-infected mice relative to Ad-Null-infected control identified as described in **(A)** are shown. Values of Ad-Null-treated controls were normalized to 100%. Values represent means ± SD *n* = 6. Differences between groups were compared using unpaired Student’s *t*-test. **p ≤ 0.01; ***p ≤ 0.001.

Together, the results in Figures 3 and 4 suggest that FXYD5 is required for LPS-induced cellular infiltration into the alveolar space. To evaluate whether the increased level of FXYD5 leads to enhanced recruitment of specific subcellular myeloid populations into the lung, mice were instilled with Ad-FXYD5 72 h prior to treatment with LPS for 24 h, and changes in leukocyte populations within the lung were analyzed by flow cytometry. After excluding doublets and dead cells, myeloid cells were identified using pan-hematopoietic marker CD45. Using the gating strategy described in the Section “Materials and Methods” and Figure 5A, no significant differences were detected in the recruitment of Ly6G^+^CD11b^int^CD24^int^ neutrophils (Figure 5B), NK1.1^+^CD11b^hi^CD24^hi^ NK cells (Figure 5C), or SiglecF^hi^CD11c^hi^ alveolar macrophages (Figure 5D) while SiglecF^hi^CD11c^low^ eosinophils (Figure 5E) were increased after infection with AdFXYD5. Additionally, we observed increased recruitment of CD11b^hi^MHCII^hi^ IMs (Figure 5F) and CD11b^hi^ MHCII^low^Ly6C^hi^ classical monocytes (Figure 5G) in the presence of higher levels of FXYD5 post-LPS challenge.

### CCR2^+^ Classical Monocytes Are Involved in FXYD5-Mediated Inflammation

**Figure 6:**
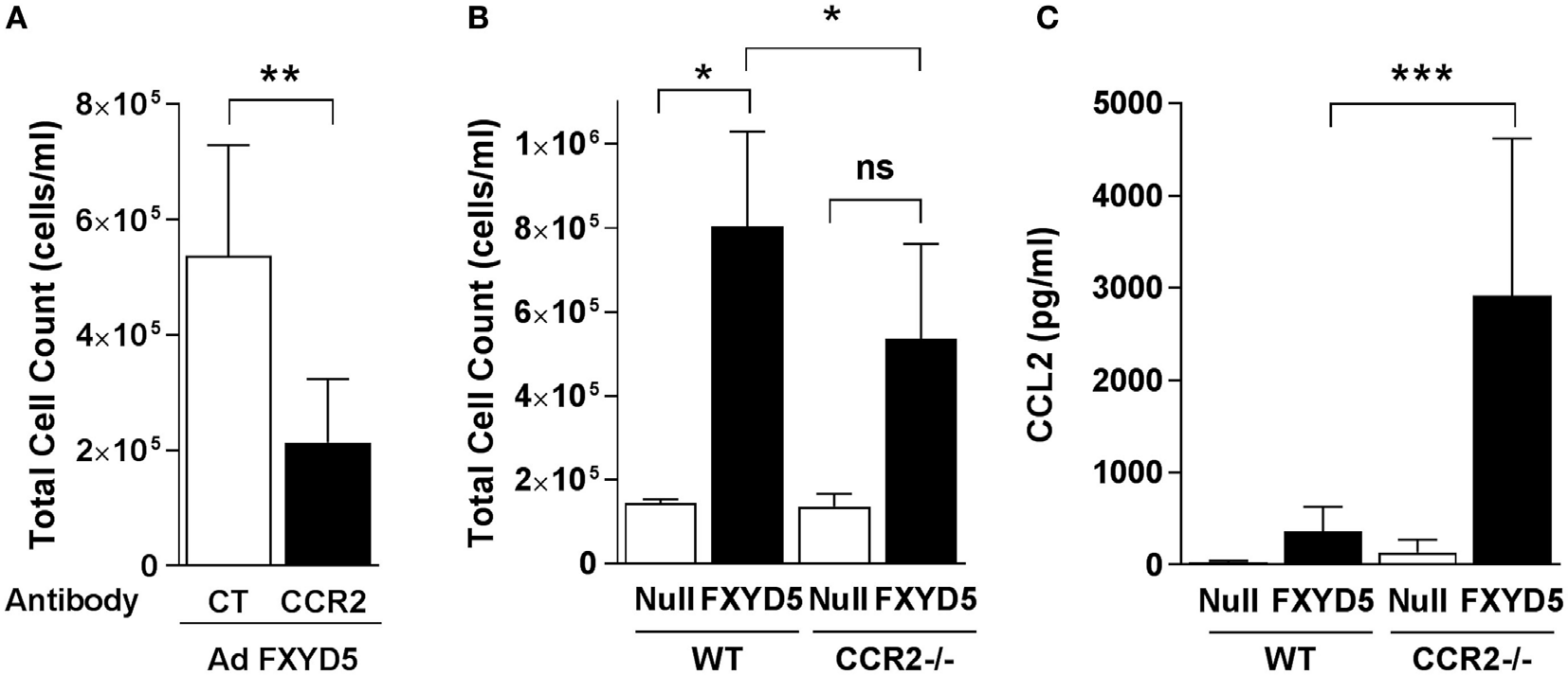
**Increased levels of FXYD5 are sufficient to recruit CCR2^+^ classical monocytes to the lung**. **(A)** Mice were instilled with Ad-FXYD5 and treated with the anti-CCR2 antibody for 24 h prior to the measurements. Total cell count in BALF was determined (*n* = 5). **(B,C)** WT and CCR2^−/−^ mice were instilled like in **(A)**. Total cell counts *n* = 3 **(B)** and CCL2 *n* = 4 **(C)** were determined in BALF. Values represent means ± SD. Differences between groups were compared using unpaired Student’s *t*-test or one way ANOVA Sidak’s multiple comparison test. *p ≤ 0.05 **p ≤ 0.01; ***p ≤ 0.001.

In mice, expression of Ly6C and CD11b identifies a subset of monocytes that expresses high levels of CCR2 (58). CCL2 and its receptor CCR2 are critical determinants for recruitment of monocytes to the lungs (4, 6, 59, 60), where they have key roles in amplifying lung injury by orchestrating an overly exuberant inflammatory response (1, 14, 61). To determine whether classical monocytes contribute to the FXYD5-induced inflammatory response, we infected mice with Ad-FXYD5 or Ad-Null, and 48 h after the infection depleted monocytes by the injection of an anti-CCR2 antibody. The presence of the antibody decreased the cellular infiltration into the lung, stimulated by infection with Ad-FXYD5 (Figure 6A). As an alternative approach, *CCR2*^−/−^ mice, which lack CCR2, and WT mice were infected with Ad-FXYD5, and BALF was collected after 72 h. The absence of CCR2 decreased the cellular infiltrates in the lungs (Figure 6B), suggesting, again, that classical monocytes play a role in FXYD5-induced inflammation. The levels of CCL2 were significantly increased in the CCR2 KO infected with Ad-FXYD5 as compared with the WT-infected mouse (Figure 6C).

Taken together, the results demonstrate that the FXYD5 abundance in AEC is increased in response to LPS, and the prevention of this increase by silencing FXYD5 partially abolishes pro-inflammatory effects of LPS. The increased levels of FXYD5 activate the production of CCL2 by AEC, which, in turn, leads to the recruitment of CCR2^+^ monocytes cells into the alveolar spaces to worsen lung injury.

### FXYD5 Is Required for NF-κB Activation Downstream of Several Cytokine Receptors

**Figure 7:**
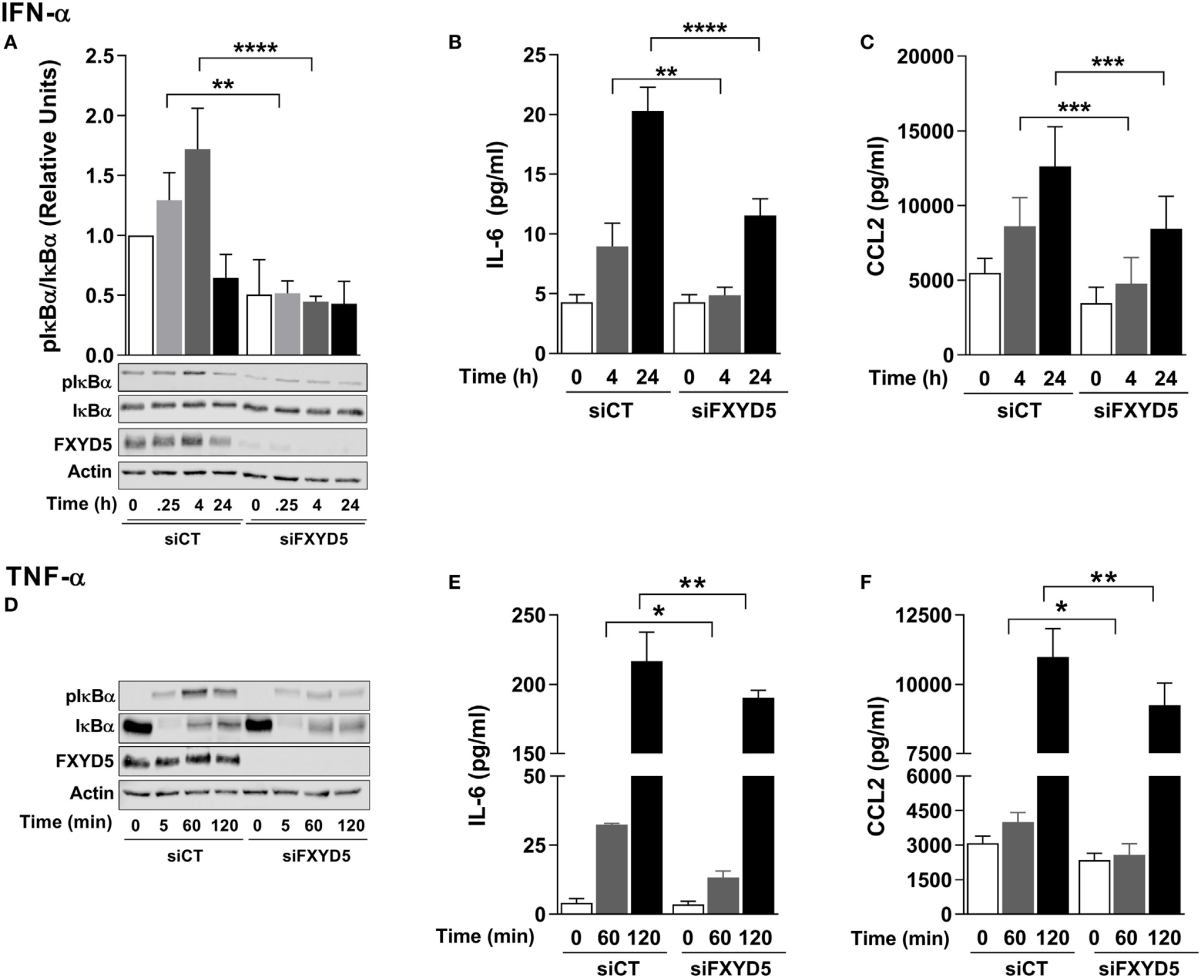
**FXYD5 is required for the NF-κB-mediated secretion of cytokines in response to IFN-α and TNF-α by alveolar epithelial cells**. **(A)** A549 cells were transfected with a control (siCT) or FXYD5 specific (siFXYD5) siRNA, 24 h later treated with 100 U/ml IFN-α for the indicated period of time, and cell lysates were analyzed by immunoblot using specific antibodies as indicated. Bottom panel: representative immunoblots. Top panel: densitometric quantification of pIκBα in relation to total IκBα *n* = 4. **(B,C)** A549 cells were treated as in **(A)**, culture media was collected, and IL-6 **(B)** and CCL2 **(C)** were determined by ELISA *n* = 3. **(D)**. A549 cells were transfected with siCT or siFXYD5, 24 h later treated with 50 ng/ml TNF-α for the indicated period of time, and cell lysates were analyzed by immunoblot using specific antibodies as indicated. Representative immunoblots *n* = 4. **(E,F)** A549 cells were treated as in **(D)**, culture media was collected, and IL-6 **(E)** and CCL2 **(F)** were determined by ELISA *n* = 4. Values of PBS-treated controls were normalized to 1. Bars represent means ± SD. Statistical significance was analyzed by one way ANOVA and Sidak’s multiple comparison test. *p ≤ 0.05; **p ≤ 0.01; ***p ≤ 0.001; ****p ≤ 0.0001, ns, non-significant.

Since expression of exogenous FXYD5 induces the inflammatory response even in the absence of LPS, we studied whether FXYD5 contributes to pro-inflammatory pathways downstream of receptors other than TLR4. To address this question, we measured the activation of NF-κB and the production of cytokines after stimulating A549 cells in the presence or absence of FXYD5 with IFN-α (100 U/ml) or TNF-α (50 ng/ml). IFN-α signals through the type I interferon receptor (IFNAR) (62), while the effects of TNF-α are initiated by its binding to the ubiquitously expressed TNF receptor 1 (TNFR1) or to the TNF receptor 2 that is mainly expressed in lymphocytes and endothelial cells (63). Treatment with IFN-α induced the phosphorylation of IκBα that was detected after 15 min and reached its maximum after 1 h (Figure 7A). The knockdown of FXYD5 prevented the activation of NF-κB and significantly inhibited the increase in cytokine secretion in response to IFN-α (Figures 7A–C).

Treatment of epithelial cells with TNF-α led to the phosphorylation of IκB and a dramatic decrease in its total amount after 5 min of treatment (Figure 7D), suggesting a rapid degradation of IκBα in these conditions. The loss of IκBα was followed by its partial recovery after 1 and 2 h of treatment (Figure 7D), which is consistent with previously published data on rapid re-synthesis of IκBα after its TNF-α-induced degradation (64). FXYD5 silencing prevented the TNF-α-induced phosphorylation of IκBα and the concomitant production of IL-6 and CCL2 (Figures 7E,F). Collectively, these results suggest that FXYD5 is a required mediator of the inflammatory response in epithelial cells.

## Discussion

The respiratory epithelium is constantly exposed to invading particles and potential pathogens. In addition to creating a barrier for pathogens, AEC secrete inflammatory mediators that recruit innate and adaptive immune cells to the alveolar space (1, 4, 7, 65–68). The mechanisms regulating the extent of epithelial inflammatory responses to infection and tissue injury are not fully understood. The data presented here demonstrate that in AEC, FXYD5, acting upstream of NF-κB, is necessary and sufficient for the secretion of pro-inflammatory cytokines *in vivo* and *in vitro*. Under our experimental conditions, LPS rapidly increases FXYD5 in AEC resulting in the secretion of CCL2 and the recruitment of CCR2^+^ monocytes to the alveolar space. These monocytes, often referred as inflammatory monocytes, are responsible for the secretion of a large number of soluble mediators that regulate the activity of other inflammatory cells.

Lipopolysaccharide-induced acute lung injury is an animal model that replicates several key pathologic processes of acute respiratory distress syndrome, including cytokine release, inflammatory cell influx, and lung capillary permeability, which results in pulmonary edema (69). In our study, LPS stimulation of isolated mouse ATII cells, as well as mouse and human alveolar epithelial cell lines, resulted in a rapid and substantial increase in the secretion of several cytokines. These effects were prevented by silencing FXYD5 using different silencing RNA, which strongly suggests a role for FXYD5 in the production of inflammatory mediators by AEC. Moreover, acute overexpression of FXYD5 in AEC increased secretion of CCL2 and IL-6. In conjunction with our previous data showing that FXYD5 overexpression produces a disruptive effect on alveolar–epithelial barrier (26), these results suggest that FXYD5 impairs the integrity of the barrier not only by directly disrupting epithelial junctions formed by Na,K-ATPase β1 subunits (26) but also by secreting cytokines that recruit immune cells into alveolar spaces, which further enhance the impairment of alveolar–epithelial barrier.

In agreement with our previous report (30), we found that inhalation of LPS results in a time-dependent increase in FXYD5 expression in the lung. This increase temporally correlated with the secretion of cytokines into the BALF and recruitment of immune cells to the lung. Either after LPS instillation or FXYD5 overexpression, we observed that a significant portion of FXYD5 is localized at the PM and heavily *O*-glycosylated (26), which contrasts with previous studies that reported that in normal tissues, including the lung, FXYD5 is expressed only as a low molecular mass protein with no or very minimal glycosylation (24, 29). Moreover, we showed that endogenous expression of FXYD5 in the lung epithelium is required for the epithelial inflammatory response, as silencing of FXYD5 decreased the number of cells in BALF after LPS treatment. This is consistent with previous reports suggesting that leukocyte recruitment during bacterial infection is due to the response of the alveolar epithelium rather than resident alveolar macrophages (8) and that the profile of cytokines released by ATII cells determines specific leukocyte recruitment (70). The data presented here demonstrate that FXYD5 overexpression in the absence of LPS or other stimuli is sufficient to activate cytokine secretion in AEC and to increase the number of cells in BALF, suggesting that the increase in FXYD5 alone, by stimulating cytokine secretion, leads to the recruitment of immune cells into the lung. The recruitment of cells by FXYD5 overexpression was decreased by treating mice with the antibody against CCR2, and the same effect was observed in mice lacking CCR2, indicating that FXYD5-induced secretion of CCL2 causes chemotaxis of CCR2-positive monocytes to the alveolar spaces.

The overexpression of FXYD5 in conjunction with LPS treatment significantly increased the recruitment of interstitial and monocyte-derived macrophages to the lung. Tissue resident alveolar macrophages are the predominant immune cells found within the alveolar airspaces during steady-state conditions, while classical inflammatory Ly6C^hi^ monocytes and IM represent a very low proportion of circulating white blood cells in an uninfected mouse and are rapidly recruited to sites of infection and inflammation (58, 71, 72). Upon stimulation, Ly6C^hi^ monocytes exit the bone marrow in a CC-chemokine receptor 2 (CCR2)-dependent manner and are recruited to inflamed tissues (58). In agreement with our data, it has been described that IM expand more rapidly in response to foreign stimuli compared with alveolar macrophages as IM are preferentially replenished from blood monocytes (72) and CCR2^+^ monocytes emigration from the bone marrow is normal during early-stage of bacterial infection of mice (58). In addition, overexpression of FXYD5 increased the recruitment of eosinophils to the lung in response to LPS. These data suggest that FXYD5 induces secretion of other cytokines/chemokines because CCL2 is not among the major chemoattractants of eosinophils such as IL-5, RANTES (CCL5), eotaxin, and others (73–76).

Further, we demonstrated that the presence of FXYD5 in AEC is required for NF-κB activation induced by LPS, TNF-α, or IFN-α as FXYD5 silencing prevented IκBα phosphorylation and reduced cytokine secretion in response to these stimuli. Moreover, overexpression of FXYD5 in the absence of any stimuli induced both IκBα phosphorylation and cytokine secretion. Taken together, these results indicate that FXYD5 is an important component of NF-κB signaling pathway. This conclusion is consistent with previously published data showing that FXYD5 overexpression in breast cancer cells induces the phosphorylation of AKT (38), which promotes the transcriptional activity of NF-κB-responsive promoter elements and increases levels of CCL2 mRNA (38, 41). Taken together, these data suggest that FXYD5 increases CCL2 transcription by inducing AKT-dependent activation of NF-κB signaling. In support of a role of an FXYD5/AKT dependent activation of NF-κB, binding of IFN-α to IFNAR activates PI3K *via* STAT5, which in turn, activates NF-κB (77–79). Activation of PI3K has been also described downstream of TLR4 and TNFR1 (80, 81). Our recent data in kidney cells, stably transfected with FXYD5, suggested that FXYD5 modulates NF-κB signaling by regulating the location of TNF-α receptor, TNFR1 (30). It is possible that the plasmalemma-located FXYD5, by interacting with the PM receptor complexes, modulates their association with other proteins as well as their location and mobility in the membrane. Such a possibility is consistent with the data showing that the efficiency of LPS/TLR4 signaling is affected by receptor mobility in the lipid bilayer that permits its clustering and binding to other proteins (82–85). However, considering significant differences in the composition of these receptor complexes as well as the fact that FXYD5 activates NF-κB even in the absence of other stimuli, a possibility that the intracellular forms of FXYD5 contribute to NF-κB signaling downstream of the PM receptors but upstream IκBα phosphorylation cannot be excluded. Taken together, our results suggest that the presence of FXYD5 in the alveolar epithelium is required for stimuli-induced pulmonary inflammation and injury. The deleterious effects of enhanced FXYD5 may be twofold: (1) the impairment of the function of the epithelial barrier through the disruption of adherens junctions (26) and (2), as shown here, the activation of the NF-κB pathway to recruit CCR2^+^ monocytes and IMs.

In conclusion, FXYD5 is a pro-inflammatory protein, which activates NF-κB-dependent cytokine secretion and infiltration of immune cells to the alveolar spaces. A better understanding of the mechanism by which alveolar epithelial FXYD5 modulates the expression of CCL2 and other cytokines may help to develop new therapies for the treatment of pulmonary inflammation following exposure to various Gram-negative bacteria commonly found in hospital settings.

## Ethics Statement

Mice were provided with food and water *ad libitum*, maintained on a 14-h-light–10-h-dark cycle, and handled according to National Institutes of Health guidelines and an experimental protocol approved by the Northwestern University Institutional Animal Care and Use Committee.

## Author Contributions

PB, PS, ET, AY, and NM performed experiments; PB assisted with the research design and data analysis; KR, HP, and JS provided reagents; PB, HP, KR, and JS discussed and edited the manuscript; OV and LD designed the research, performed experiments, analyzed data, and wrote the manuscript.

## Conflict of Interest Statement

The authors declare that the research was conducted in the absence of any commercial or financial relationships that could be construed as a potential conflict of interest.
